# Polymicrobial Prosthetic Valve Endocarditis Due to Neisseria gonorrhoeae and Pseudomonas fluorescens in a Patient With Tetralogy of Fallot: A Case Report

**DOI:** 10.7759/cureus.27677

**Published:** 2022-08-04

**Authors:** Matthew K Edwards, Milan B Bhattacharya, Shane Clark, Lennox K Archibald, Gautam S Kalyatanda

**Affiliations:** 1 Department of Medicine, Case Western Reserve University School of Medicine, Cleveland, USA; 2 Department of Medicine, University of Florida College of Medicine, Gainesville, USA; 3 Department of Infectious Diseases and Global Medicine, University of Florida College of Medicine, Gainesville, USA

**Keywords:** tetralogy of fallot, polymicrobial endocarditis, neisseria gonorrhoeae, infective endocarditis, pseudomonas fluorescens

## Abstract

Disseminated gonococcal infections are rare clinical entities and a few progress to endocarditis. Endocarditis caused by Pseudomonasis even more infrequent, with the few reported cases associated with either intravenous drug use, prosthetic heart valves, or pacemakers. We report a case of a 25-year-old male patient with Tetralogy of Fallot presenting with anasarca and diagnosed with endocarditis due to *Neisseria gonorrhoeae* and *Pseudomonas fluorescens*. To our knowledge, this is the first case of tissue-proven infective endocarditis due to *P. fluorescens* with concomitant *N. gonorrhoeae* bacteremia. Clinical management of polymicrobial endocarditis in young adults includes obtaining a detailed sexual history, using multiple diagnostic methods to confirm endocarditis, and promptly initiating broad-spectrum antibiotic therapy.

## Introduction

Infective endocarditis (IE) is a rare occurrence in the post-antibiotic era, with an incidence of 4.6/100,000 [1]. Both hospitalization rates and IE incidence have risen steadily over the past two decades, with the greatest increases occurring in older men [1]. Patients with underlying heart disease comprise 67% of IE cases, with 45% of patients having prosthetic valves and 4.5% having a congenital heart disease such as Tetralogy of Fallot [1]. Tetralogy of Fallot is the co-occurrence of congenital right-sided ventricular outflow tract obstruction and hypertrophy, ventricular-septal defect, and overriding aorta. Tetralogy of Fallot predisposes a patient to right-sided endocarditis due to altered cardiac hemodynamics and the required corrective procedures, including the placement of an artificial pulmonic valve [2]. Right-sided IE accounts for 5-10% of all IE cases and is most frequently associated with intravenous (IV) drug use, in situ cardiac devices, and congenital abnormalities [3].

Staphylococcus and Streptococcus are the most common etiologic agents implicated in IE [1]. Gram-negative bacteria, including *Neisseria gonorrhoeae* and Pseudomonas, are much less frequent (6.5%) causes of IE [1,4]. Although *N. gonorrhoeae* is a common cause of sexually transmitted infection in the United States, gonococcal endocarditis is a rare and life-threatening complication that occurs in nearly 2% of patients with disseminated gonococcal infection [5]. A notable resurgence in cases of gonococcal endocarditis described in the recent literature may correspond with a rise in antimicrobial resistance [1]. Endocarditis caused by Pseudomonas is less common and is associated with intravenous (IV) drug use (>90% of cases), prosthetic heart valves, and pacemakers [4,6]. Endocarditis due to a singular or co-infection with *P. fluorescens* has not been previously described in the medical literature.

We report a case of a patient diagnosed with a co-infection of the endocardium by *N. gonorrhoeae* and *P. fluorescens* and review the literature on IE caused by these bacteria.

## Case presentation

A 25-year-old male presented to the emergency department with a one-week history of anasarca. The patient reported chills, night sweats, intermittent fevers, loss of appetite, productive cough, one episode of vomiting, and midline chest pressure worsened by inspiration over the past month. He denied any dysuria, genital lesions, or palpitations. His medical history was significant for Tetralogy of Fallot, with a complete intracardiac repair in 1998, pulmonic valve replacement in 2006, and right ventricular outflow patch repair in 2012, all without complications. The patient reported frequent use of recreational marijuana but no history of injection drug use. He had a history of gonorrheal urethritis treated with antimicrobials three years prior and noted recently having unprotected sex with two partners, one of whom was treated for gonorrhea a few weeks before his symptoms began.

The patient had a fever of 104.4 °F, tachypnea, and tachycardia on presentation. A physical examination revealed evidence of decompensated heart failure and volume overload, including bilateral crackles throughout the lungs, a grade IV holosystolic murmur, tender hepatomegaly, and generalized anasarca. There was no evidence of rash, penile discharge or ulceration, tendinopathy, or arthropathy. Laboratory findings demonstrated an elevated leukocyte count with a neutrophil predominance, microcytic anemia, and a high creatinine, C-reactive protein, and erythrocyte sedimentation rate (Table 1).

**Table 1:** Notable laboratory results

Laboratory parameters (units)	Patient values	Reference range
Leukocytes (cells/L)	14.0 x 10^9^	4.0-10.0 x 10^9^
Neutrophils (cells/L)	11.0 x 10^9^	2.0-8.0 x 10^9^
Hemoglobin (g/dL)	5.5	12.0-16.0
Creatinine (mg/dL)	1.6	0.7-1.4
C-reactive protein (mg/L)	86.4	0.0-10.0
Erythrocyte sedimentation rate (mm/hour)	>130	<15

**Figure 1:**
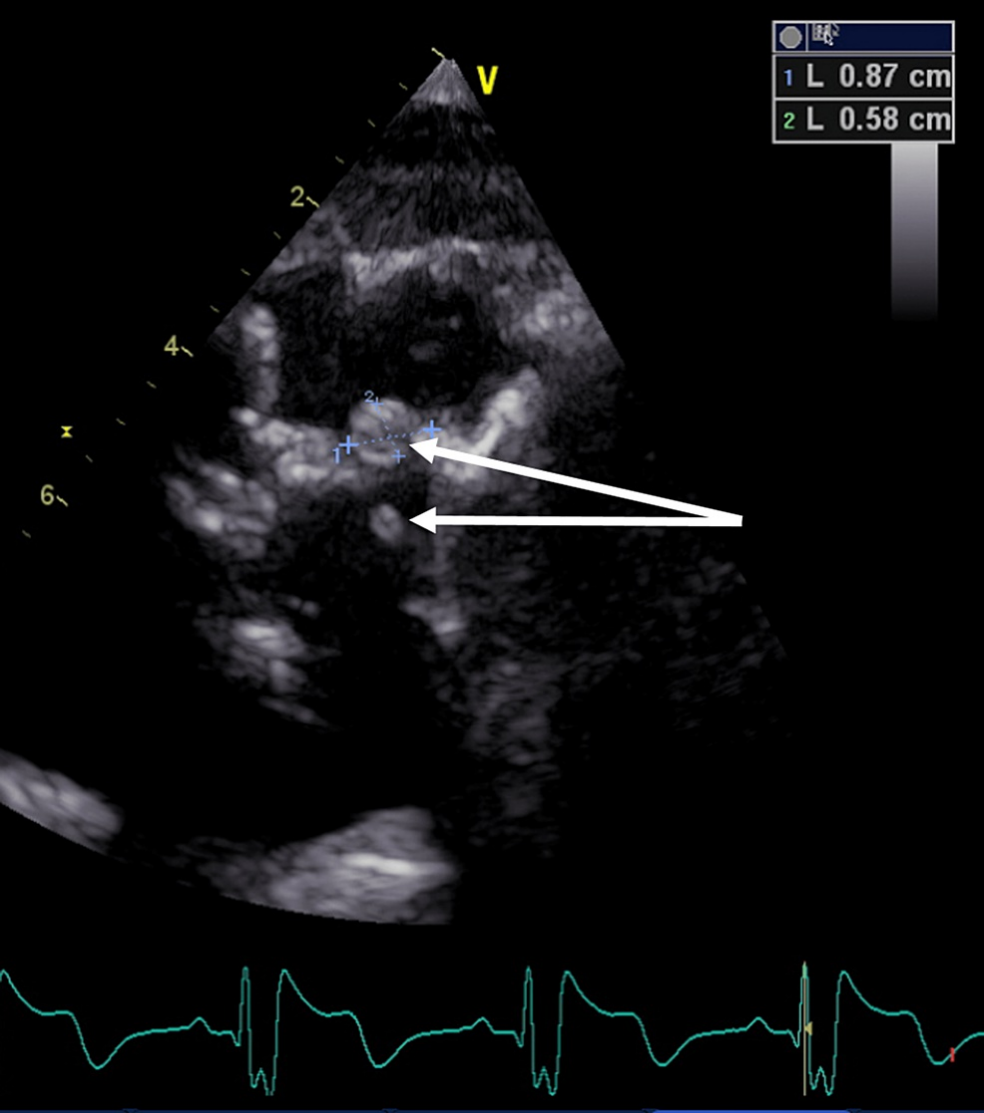
Transthoracic echocardiogram/short-axis view at the basal level White arrows showing pulmonic valve vegetations

A transthoracic echocardiogram showed a moderate decrease in right ventricular function, mild decrease in left ventricular function, and possible vegetations on the pulmonary valve (Figure 1).

The patient was transfused with two units of packed red blood cells and started on vancomycin and 2 g of IV cefepime was administered every eight hours. Given his elevated creatinine and marked edema, he was started on gentle diuresis with furosemide. On the second day of admission, a serum fourth generation antigen/antibody assay for human immunodeficiency virus (HIV), QuantiFERON TB Gold (a test to detect tuberculosis infection), and *Chlamydia trachomatis-Neisseria gonorrhoeae* nuclear amplification assay (NAA) in urine were all negative. However, blood cultures yielded a pan-sensitive strain of *N. gonorrhoeae* in one out of four bottles. The patient’s antimicrobial regimen was therefore narrowed to ceftriaxone, after which three subsequent surveillance blood cultures returned negative. On hospital day ten of admission inflammatory markers started to rise, increasing concern for worsening endocarditis.

On day 14 of admission, the patient successfully underwent surgery for a right ventricular to pulmonary artery replacement with the reconstruction of the pericardium. He was extubated the following morning and had an uncomplicated postoperative course. Intraoperative biopsies of the endocardial tissue grew *P. fluorescens* on two separate specimens at 24 hours of incubation. Identification of this bacteria was performed with bioMerieux VITEK2 (VITEK® 2, bioMerieux, USA) and confirmed by Matrix Assisted Laser Desorption Ionization Time of Flight (MALDI-TOF). The VITEK2 antibiogram demonstrated susceptibility to amikacin, cefepime, ceftazidime, ciprofloxacin, gentamycin, levofloxacin, meropenem, piperacillin/tazobactam, tobramycin, and resistance to trimethoprim and sulfamethoxazole. His antimicrobial regimen was therefore transitioned back to cefepime with a parallel improvement in symptoms and inflammatory markers. He was discharged on day 23 following admission with a plan for six weeks of treatment with cefepime and scheduled follow-up outpatient clinic appointments with Cardiology and Infectious Disease services.

He was seen in the Infectious Disease clinic after completion of his antibiotic course and was recovering well.

## Discussion

Gonorrheal infections are increasingly common in the United States, with over 616,000 reported cases in 2019 alone, representing a 92% increase since 2009 [7]. Disseminated gonococcal infections are relatively uncommon, occurring in approximately 0.4-3% of these patients, with only 1-2% of those progressing to endocarditis [8,9]. Though much less common now, gonococcal endocarditis in the pre-antibiotic era comprised 11-26% of all cases of endocarditis and was almost always fatal [5].

Patients with *N. gonorrhoeae* endocarditis are predominantly sexually active men between 15 to 35 years of age, the demographic group that has had the greatest recent rise in *N. gonorrhoeae* infection rates [7,10]. Conversely, women are more likely to have asymptomatic gonococcal infections and may be an underdiagnosed demographic for IE [11]. Few patients experience genitourinary symptoms typical of a sexually transmitted infection (STI) before seeking medical care and most lack a history of valvular heart disease [5,10]. Symptoms include fever, chills, dyspnea, malaise, tachycardia, murmurs, arthritis, and systemic embolization and usually develop within two-four weeks [12]. Cases typically involve damage to native valves, with the aortic valve comprising 50-78% of cases and the mitral valve comprising 24% [5,9,12]. Pulmonary valve involvement, as seen in this patient, is significantly less common [12]. Prosthetic valve involvement is rare; of 39 reported *N. gonorrhoeae* cases, only two patients (5%) had infections of prosthetic valves [9]. Of note, these data were published four years prior to the discovery of the first gonococcal strain with ceftriaxone resistance and high-level azithromycin resistance [13]. The low occurrence of prosthetic valve involvement reported in the literature may be due to the young age and lack of valvular heart disease present in the demographic group most likely to acquire gonorrhea. However, with the rising incidence of drug-use-associated IE and subsequent treatment with prosthetic valve placement, this younger population may be at an increased risk and thus worthy of closer monitoring [14]. The patient presented here is atypical in this regard due to his history of Tetralogy of Fallot and prosthetic valves.

Positive blood cultures and the presence of valvular vegetations are important case findings for diagnosing gonococcal endocarditis. One study found that 96% of gonococcal endocarditis cases had positive blood cultures and 95% had identified vegetations on valves [12]. 

Antimicrobial resistance is a significant concern for the treatment of *N. gonorrhoeae*; recent surveillance data by the World Health Organization found that 84% of reporting countries observed increased resistance to azithromycin, 31% observed increased resistance to ceftriaxone, and 100% observed resistance to ciprofloxacin [15]. National surveillance data by the U.S. Centers for Disease Control from 2018-2019 showed 11% of isolates demonstrating azithromycin resistance and 0.5% demonstrating ceftriaxone and cefixime resistance [16]. There are few data available demonstrating antibiotic resistance in *N. gonorrhoeae* endocarditis. In a review of 28 gonococcal endocarditis cases, resistance to ciprofloxacin was found in 7% of cases and penicillin resistance was seen in 20% of cases [11]. In the event of antimicrobial resistance limiting therapeutic options, the mortality rate would likely be much higher than the 18.6% rate seen in gonococcal endocarditis treated with appropriate antibiotics and surgery [12].

Compared to *N. gonorrhoeae*, *P. fluorescens* is a much less common cause of infections, which occur predominantly in immunocompromised patients and via water exposure [17,18]. *Pseudomonas fluorescens* rarely causes invasive hospital-acquired infections, with most iatrogenic bacteremia attributable to either the transfusion of contaminated blood products or the use of contaminated equipment for IV infusions [19]. While multiple cases of *P. aeruginosa* endocarditis have been published, *P. fluorescens* has never been reported as a cause of endocarditis, underscoring its novelty as a co-infective organism in this case. The patient in this case was diagnosed with gonococcal endocarditis based on one of four blood cultures that grew *N. gonorrhoeae* and vegetation seen on transthoracic echocardiogram. Given the timing of the patient’s symptoms and his exposure to sexual activity, the *N. gonorrhoeae* positive culture was considered significant. Cultures failed to reveal *P. fluorescens *in the bloodstream, however this organism was isolated on multiple tissue specimens from the endocardial tissue, making it less likely that this was a contaminant. Our patient received IV cefepime therapy for four days, which possibly decreased the yield of detection for *P. fluorescens* in the blood cultures. It is therefore unclear if the patient acquired the infection prior to a hospitalization or if this represents a nosocomial infection.

## Conclusions

This patient’s initial presentation was symptomatically typical of gonococcal endocarditis and the diagnosis was expedited by taking a thorough sexual history. Obtaining an accurate sexual history for patients presenting with concerns consistent with endocarditis may be complicated by the typical patient demographic group and the rarity of association between STIs and this disease. Given the rapid treatment of this patient’s endocarditis and his positive outcome, this case serves as a valuable example of the clinical approach to polymicrobial endocarditis. Using multiple diagnostic methods to confirm endocarditis, treating with broad-spectrum antibiotics throughout, and obtaining a detailed sexual history and social history can all improve patient outcomes.
